# Mental health literacy amongst children with common mental health problems and their parents in Java, Indonesia: a qualitative study

**DOI:** 10.1017/gmh.2022.5

**Published:** 2022-02-21

**Authors:** Helen Brooks, Benny Prawira, Kirsten Windfuhr, Irman Irmansyah, Karina Lovell, Armaji Kamaludi Syarif, Suzy Yusna Dewi, Swastika Wulan Pahlevi, Atik Puji Rahayu, Syachroni, Annisa Rizky Afrilia, Laoise Renwick, Rebecca Pedley, Soraya Salim, Penny Bee

**Affiliations:** 1 Division of Nursing, Midwifery and Social Work, School of Health Sciences, Manchester Academic Health Science Centre, University of Manchester, Manchester, UK; 2 Atma Jaya Catholic University of Indonesia, Jakarta, Republic of Indonesia; 3 Into the Light, Jakarta, Republic of Indonesia; 4 NHS Benchmarking Network, Manchester, UK; 5 National Institute of Health Research and Development, Ministry of Health, Jakarta, Republic of Indonesia; 6 Greater Manchester Mental Health Foundation Trust, Manchester, UK; 7 Soeharto Heerdjan Mental Hospital, Jakarta, Republic of Indonesia; 8 Soerojo Mental Hospital, Magelang, Republic of Indonesia; 9 Marzoeki Mahdi Mental Hospital, Bogor, Indonesia; 10 Pulih@the Peak – Women, Youth and Family Empowerment Centre, Jakarta, Republic of Indonesia

**Keywords:** Children and young people, Indonesia, mental health literacy, middle-income country, qualitative research

## Abstract

**Background:**

Optimising mental health literacy (MHL) at the individual and population level can be an effective mental health improvement and prevention tool. However, concepts of MHL are largely based on evidence from high-income countries. Little is known about the manifestation and role of MHL in countries where collectivist health and social cultures are dominant.

**Aim:**

This study aimed to examine the MHL of Indonesian children and young people (CYP) with experience of common mental health problems and their parents.

**Methods:**

Semi-structured interviews with 40 participants (19 CYP aged 11–15 with experience of common mental health problems and 21 parents) from three areas of Java, Indonesia. Data were analysed using framework analysis, informed by Jorm's 1997 Mental Health Literacy Framework.

**Results:**

Parents and CYP demonstrated relatively low levels of MHL defined from a conventional perspective. Religiosity and spirituality were salient in participants' accounts, particularly parents, as were narratives about personal responsibility. These beliefs appeared to contribute to a high level of self-blame for mental illness, self-reliance for symptom management, the foregrounding of support from spiritual/traditional healers and a reduced propensity to access professional help. CYP were heavily reliant on family support, but parents often felt they were not best placed to communicate with their children about mental health. Providing trusted, technology-based sources of mental health information were advocated by CYP.

**Conclusion:**

Robust efforts are needed to improve MHL in low- and middle-income countries drawing on culturally appropriate approaches to reduce stigma and optimise timely, effective help-seeking for CYP. Enhancing parental and family level literacy may be efficacious, especially when combined with mechanisms to facilitate open communication, as may the development of standalone interventions directly developed to reach younger generations. Future research may usefully establish the comparative efficacy and acceptability of these different approaches.

## Introduction

Indonesia, a lower middle-income country in southeast Asia, is the world's fourth most populous country with 23.5 million youths aged 13–17 (approximately 9% of the total population; World Health Organisation, 2014). Mental disorders account for approximately one-quarter of the disease burden amongst young Indonesians and suicidal behaviour remains a significant problem (Mokdad *et al*., 2016; World Health Organisation, 2017). In the 2018 Basic Health Research Survey in Indonesia (*Riskesdas*), 6.2% of young people aged 15–24 were found to be suffering from depression and 10% with emotional disorders (Badan Penelitian dan Pengembangan Kesehatan, 2019). The treatment and prevention of non-communicable diseases, promotion of mental well-being and innovative approaches targeted at addressing the impact of depression are global imperatives (World Health Organisation, 2008). In particular, an emphasis on community and population level prevention and promotion initiatives is posited as an effective approach to mental health improvement, particularly in low-resource settings where specialist mental health service provision is limited or underdeveloped.

Mental health literacy (MHL), originally defined by Jorm and colleagues, encompasses a cluster of skills across four broad domains (Jorm *et al*., 1997): (1) recognition of mental illness, (2) understanding the causes of mental ill health, (3) help seeking knowledge (self-help strategies, professional help seeking and seeking mental health information) and (4) beliefs about facilitators and barriers to help seeking (Jorm *et al*., 1997). This definition of MHL has further developed since its original inception to also incorporate positive mental health, stigma and self-management self-efficacy (Kutcher *et al*., 2015; Kutcher *et al*., 2016; Jorm, 2020).

However, research into MHL in children and adolescents in low- and middle-income countries (LMICs) has received comparatively less attention (Jorm, 2012; Jacob and De Guzman, 2016; Bale *et al*., 2020; Mansfield *et al*., 2020). Notable exceptions are the two recent reviews of the extant literature on youth MHL and conceptualisations of positive mental health and wellbeing which identify key areas of focus including: greater understanding of common mental health problems; positive health promotion to strengthen autonomy and empathy and better knowledge of mental health support (Renwick *et al*., 2021, Renwick *et al*., forthcoming). Evidence also suggests that increasing MHL is an effective mental health improvement and prevention tool (Seedaket *et al*., 2020) and children and young people (CYP) consistently express a desire for additional information and support in relation to mental health (Riebschleger *et al*., 2019). Such interventions may have particular salience in the Indonesian context given recent evidence which suggests poor MHL is pervasive amongst CYP (Willenberg *et al*., 2020; Brooks *et al*., 2022).

The development of youth MHL assessment scales and a child MHL model is arguably indicative of a burgeoning focus on MHL amongst CYP populations (Campos *et al*., 2016; Bale *et al*., 2020; Zenas *et al*., 2020). However, despite calls for a life-course approach to MHL to ensure that conceptualisation, intervention development and assessment are developmentally appropriate (Kutcher *et al*., 2016), a gap in the literature remains. Few studies have taken in-depth qualitative approaches which are likely to be fundamental to developing tailored and culturally appropriate^1^ MHL interventions (Leighton, 2010; Mansfield *et al*., 2020).

There is growing recognition of the impact of cultural concepts, values and beliefs on the expression, experience and treatment of mental health difficulties (Bhugra *et al*., 2021). The concept of MHL, however, remains largely based on western ideas of mental health developed from adult populations living in high-income countries. In Indonesia, recent studies have explored dominant mental health and illness beliefs amongst CYP including the central role of religion in Indonesian culture (Brooks *et al*., 2022), MHL amongst specialist and lay workers (Praharso *et al*., 2020) and broader conceptualisations of mental health and illness amongst older Indonesian adolescents (16–18 years) (Willenberg *et al*., 2020). These emerging studies illustrate the central importance of adopting a culturally appropriate approach to MHL to optimise future intervention programmes.

Systematic reviews highlight the role of lay support networks and parents in the acquisition of MHL, particularly in LMIC contexts characterised by collectivist/communalistic cultures (Renwick *et al*., 2021; Renwick *et al*., forthcoming). Yet, research exploring MHL within its socio-cultural context, from the perspective of young people with lived experience of mental illness and their parents, is lacking. Tailoring our understanding of MHL culturally, developmentally and experientially is important to inform effective interventions and address wider social issues relating to stigma (Ng, 1997). As such, this comparative study aimed to qualitatively examine the MHL of Indonesian CYP with lived experience of mental illness and their parents. It is important to note that personal experience of mental health problems either directly or through close personal connections it thought to be associated with increased levels of MHL (Angermeyer *et al*., 2004; Furnham and Blythe, 2012; Lam, 2014; Riebschleger *et al*., 2019).

This study forms part of a larger programme of research study which is designed to co-produce and evaluate an MHL intervention for young people aged 11–15 in Indonesia, The IMPeTUs study (Brooks *et al*., 2019*a*; Brooks *et al*., 2021).

## Methods

This study utilised a qualitative explorative design incorporating semi-structured interviews. Ethical approval for the study and all documented procedures was granted by the University of Manchester Research Ethics Committee (Ref: 2018-4949-7908) and The Ministry of Health, Indonesia (Ref: LB:02.01/2/KE.201/2019).

### Participants

Participants were recruited from three areas in Java, Indonesia: Jakarta, Magelang and Bogor. Study areas were chosen due to differing levels of culture, urbanisation and health service development (Brooks *et al*., 2019*a*).

Inclusion criteria for both stakeholder groups were as follows:

- CYP: children or young people aged 11–15 with experience of anxiety and depression.
- Parents: parents of children or young people aged 11–15 with experience of anxiety and depression.

These disorders were chosen as they constitute high prevalence conditions in Indonesia and represent two of the leading causes of disease burden globally (Jane Costello *et al*., 2006). Depression and anxiety often co-exist; ameliorating the negative impact of depression is an explicit priority of Indonesia's National Mental Health Action Plan and international sustainable development goals (World Health Organisation, 2015). In order to recruit CYP with a diagnosis of anxiety and depression, direct invitations were sent out through primary care services and child and adolescent mental health services (CAMHS) to parents of eligible CYP. Posters advertising the study were also displayed in relevant health service buildings.

Eligible parents contacted the research team directly to express an interest in the study and were provided with adult and child friendly, illustrated versions of participant information sheets and parental consent forms. CYP could only participate in the study once verbal assent was provided by the young person and written consent for the young person's involvement was provided by parents or relevant carers and witnessed by a third party. Participants were purposively sampled in relation to gender, geographical location and age. A sample of 40 participants was recruited to the study: 21 parents and 19 CYP (see Table 1). Two children (whose parents were involved) did not wish to take part in an interview.

**Table 1.** Demographic information

	CYP (%) (*n*)^a^	Parents (%) (*n*)
Gender		
Male	63% (12)	14% (*n* = 3)
Female	37% (7)	86% (*n* = 18)
Age		
Mean	12.8	42.15
Range	11–15	24–60^b^
Area		
Jakarta	26% (5)	29% (6)
Bogor	32% (6)	33% (7)
Magelang	42% (8)	38% (8)

a All CYP participants had a diagnosis of anxiety or depression.

b Age for one parent was not provided.

### Data collection

At the point of expressing an interest in study participation, an initial meeting was arranged between a member of the research team, the CYP and their parent/carer. During this meeting, the process of taking part in the research was explained and participants were provided with adult and child friendly versions of information sheets. Parents/carers were also provided with a consent form and all parties were given the opportunity to ask any questions relating to participation before a date was arranged for the interviews. Separate semi-structured qualitative interviews with CYP and parents were undertaken in private rooms in health service or research organisations by members of the research team who had all received training and ongoing supervision in qualitative data collection.

Semi-structured interviews were conducted which explored beliefs, attitudes and experiences of mental health with a specific focus on anxiety and depression. Priorities for intervention were also examined. Interview schedules were structured around the components of MHL covering recognition of mental illness, knowledge about causal factors, perceptions of self-help strategies and views on professional, informational and lay support options (Jorm *et al*., 1997).

Interviews lasted between 20 and 40 minutes, were conducted in Bahasa Indonesian and were recorded digitally before being transcribed verbatim. Transcripts were anonymised at the point of transcription before being translated into English by a member of the research team for the purposes of analysis. 5% of transcripts was checked by II to ensure accuracy of translation.

### Data analysis

Data from CYP and parents were analysed separately using a framework approach which incorporated both inductive and deductive coding (Gale *et al*., 2013). The first phase involved reading and re-reading allocated transcripts in order for data analysts to familiarise themselves with the data. The second stage involved coding aspects of participants' narratives to one of the MHL components. This was undertaken manually using Excel with data from CYP and parents coded to different Excel sheets.

Coded segments of transcripts were also tagged to memos which linked data excerpts to emerging analytical observations and propositions within Excel. HB and KW independently analysed the first four transcripts before meeting to ensure consistency of coding by comparing and discussing interpretations and coding decisions. The remaining transcripts were then coded to allow for illustration within the manuscript through the provision of raw-data segments to support descriptions of interpretations.

MHL components within individual datasets were then coded inductively by HB, KW and BP to develop overarching categories within components and transcripts were revisited as a whole to consider any data that fell out with the MHL framework. No relevant omitted data were identified during this process. Finally, links between components were considered, and differences between the data collected from CYP and parents were systematically examined.

Data collection was undertaken by Indonesian researchers who had neither prior experience of MHL interventions nor any existing relationships with research participants. Analysis was undertaken by HB, BP and KW. The research process was closely supervised by II, HB and PB who have published in the area of mental health in Indonesia and are leading an MRC study to co-develop and undertake case study evaluations of a depression and anxiety-focused MHL intervention (Brooks *et al*., 2019a). The study was developed on the basis of the value of MHL interventions to support the mental health and wellbeing of CYP.

Data and preliminary analysis were presented to the Patient and Public advisory group and other relevant stakeholders (parents, teachers, health professionals and CYP) at a four day workshop in January 2020 to further verify interpretations and ensure the analysis was grounded in the lived experience of mental health problems in Indonesia.

## Results

A table presenting exemplar quotes for key points of convergence and divergence between parent and CYP views mapped to the MHL framework used to theoretically underpin the study can be found in Appendix 1.

### Ability to recognise different forms of stress/emotional distress/mental illness

Both children and parents demonstrated relatively limited awareness of formal diagnostic categories. Anxiety was more accurately described with less understanding apparent relating to depression. There was less awareness of other types of mental health conditions. Anxiety was typically described in terms of transient, physical sensations of fear, while depression was described on a more abstract level, with greater negative connotations, including pervasive or more prolonged impairments in concentration, focus or daily function.

Narratives about mental health difficulties from both groups appeared to differentiate between common mental health problems and more severe forms of mental illness on the basis of severity and behavioural presentation. Parents described their children's difficulties more commonly as these less serious types of mental health problems rather than severe mental illnesses. This demonstrates a degree of normalisation of common mental health problems amongst study participants.

There was a sense amongst both groups that mental illness, particularly more severe forms of mental illness was a form of ‘abnormality’ and stigmatising language was often used to describe people with mental illness.

Children demonstrated little awareness or recognition of their own mental health problems and often described having to be told they were unwell by parents or professionals. Parents on the other hand clearly described observing the deterioration in their child's mental health in terms of timings and key developmental events. CYP looked to parents in order to develop their MHL but parents did not appear to gain learning about mental health from their child's direct experiences. Where parents demonstrated greater ability to recognise mental illness, this appeared to be related to their own lived experience rather than their children's experiences. However, this increased recognition did not necessarily translate to the MHL of their child.

There was also a divergence in terms of the indicators of mental illness attributed by CYP and parents. Parents tended to focus on behavioural indicators such as fighting or aggression whereas CYP were more likely to focus on feelings and emotions with some lower recognition of behavioural indicators.

### Causes or risk factors for mental health problems

The causes or risk factors attributed to mental illness were similar across both groups of participants and broadly fell into three sub-themes: biological and developmental causes, social and relational causes and environmental causes. Mental illness was often associated with deficiencies of character or poor lifestyle choices (including a lack of religion) amongst CYP and to a lesser extent parents (who tended to blame themselves) which underpinned additional causal factors.

Compared to parents, there was more variation in the responses from CYP. While some CYP were unaware of the causes of their mental illness other CYP identified multiple interacting factors and that these could vary based on individual circumstances. This appeared to be as a result of their own experience of mental health problems.

#### Biological and developmental causes

There was recognition amongst both CYP and parents of the biological origins of mental illness. While both CYP and parents described the genetic heritability of mental illness, parents also described stages of development when mental illness manifested itself in CYP. The importance of the mind–body connection was prominent in parents' accounts through the somatisation of mental health problems, particularly those who had personal experiences of mental illness.

#### Social and relational causes

Parents often identified poor parenting or dysfunctional family relationships as a cause of their child's mental illness; this was less often identified by CYP. Descriptions of these events were often accompanied by undertones of blame and self-blame, particularly amongst parents.

Parents and CYP coalesced in their identification of trauma as a common feature of the social causes of mental illness. This was most often described as a consequence of domestic experiences, e.g. physical abuse, sexual abuse, abandonment, but also as a result of trauma experienced outside of the family environment.

School was commonly the setting described as the most stress inducing, particularly in CYP accounts of bullying from peers or punishments by teachers. Difficult non-familial relationships with and low levels of MHL amongst peers and teachers were also observed as contributors to mental illness in CYP by both groups. Peers were often observed as a negative influence, while difficult relationships with teachers could exacerbate already difficult circumstances.

#### Environmental causes

Mental illness was frequently described as a ‘test from God’ by both CYP and parents. In contrast, a lack of religiosity was more often attributed as a cause of mental illness by parents. Less often, CYP identified mystical causes of mental illness although this was not a dominant theme amongst all CYP and was not particularly salient in parental accounts.

### Help-seeking knowledge

#### Knowledge and beliefs about self-help interventions

A focus on parental and individual responsibility for self-management was apparent in interviews with parents and to a lesser extent in CYP interviews.

Both CYP and parents foregrounded engagement with valued activities such as exercise, arts/music, playing sports, watching TV, playing games and using the internet as important self-help strategies. Helping others was also considered an important way to manage mental health problems. These activities were considered important in terms of distraction from or avoidance of current difficulties. They also provided a sense of purpose and hope for the future. Parents appeared to place increased salience on strategies that had worked previously for them. Despite this shared value attributed to engaging in valued activities, parents were more likely to identify reasons that their children did not want to or were not able to engage in such activities.

Religious and spiritual activities were particularly salient as self-help strategies in parental accounts which included praying, going to church or mosque, participating in religious practices or undertaking spiritual/traditional healing. These activities were less apparent in CYP accounts. Parents acknowledged that while religiosity helped them, their children were less inclined to want to participate in these types of activities and derived benefit from other activities as described above.

Positive relationships with and support from friends and family (and to a lesser extent counsellors, teachers, siblings and grandparents) were considered important in terms of self-management. Of particular value was talking with these lay network members about their mental health problems. Interestingly, while CYP often placed high value on parents in terms of self-management there was some divergence in parental views. While most parents felt adults were more experienced and therefore a more reliable source or support in relation to self-help, a view often endorsed by CYP, some felt that their children might be reluctant to speak to them or other adults about their mental health difficulties.

In a small number of cases, family and friends were considered by CYP to contribute negatively to self-help strategies where relationships were difficult, overwhelming or when past experience had reduced confidence in their ability or willingness to help. This was rarely acknowledged by the parents interviewed beyond the potential for some friends to be bad role models or perpetrators of bullying.

#### Knowledge of available professional help

On the surface, there was broad convergence amongst CYP and parents that professional help was useful both for direct support for CYP's mental health problems, but also for parents in understanding how best to support their child. There was general agreement that professionals provided two primary functions: providing formal therapy and the prescription of medication.

However, some parents and CYP were equivocal about the utility of professional help. Where knowledge and previous experience were minimal there was initial scepticism about how useful professional help could be. In contrast, where there had been previous positive experiences either with treatment for their child's mental health problems or their own mental health problems, belief in professional support was more positive.

Despite the value attributed to professional support, only a minority of parents actually considered obtaining support from formal health services in the first instance. Parent's conceptualisations of professional support extended to spiritual or religious healers who were often prioritised in terms of help seeking. Spiritual healers functioned as a source of support as well as a source of information about professional help seeking when parents lacked this knowledge. For one parent, perceived financial barriers to professional help seeking appeared to contribute in part to them seeking support in the first instance from their spiritual healer. Interestingly, where formal health services needed to demonstrate results to improve confidence in their utility, trust appeared inherent in relationships with spiritual and religious leaders.

#### Knowledge of how to access mental health information

Both CYP and parents coalesced in the value they attributed to different sources of mental health information. The main divergence in accounts was that CYP were more heavily reliant on family members and technology for mental health information whereas parents tended to have a greater breadth of resources to draw on and therefore cited a wider range of mental health information resources.

In general, both CYP and parents acknowledged the value of technology-based information sources including the internet, Google, social media and online games. Social media interactions were considered to provide information on mental health conditions but also self-management. Applications such as WhatsApp were also useful for sharing information relating to mental health. Some parents, however, felt less skilled in using technology to seek information so therefore relied on other sources, e.g. professionals, books and trusted friends/family. Religious sources of information were considered relevant by both CYP and parents but was especially apparent in parental accounts. Other sources of information included health professionals, TV, films and books.

### Barriers and facilitators to help-seeking

#### Community awareness, negative attitudes and stigma

Both parents and CYP acknowledged that it was important to seek help. However, they also acknowledged that they often avoided seeking help from others as they anticipated negative attitudes and stigma from others including close and extended family members, health professionals, those in school settings and the wider community. This was attributed to low levels of understanding about mental health in Indonesian generally.

Participants acknowledged that knowledgeable and non-judgemental friends, family and wider community members could facilitate help-seeking through providing advice and signposting.

#### Personal experience of and confidence in help-seeking

CYP often described lacking confidence to express their emotions and share their stories in order to facilitate help-seeking as well as expressing concerns about being believed. For several parents and CYP their current service contact was the first time they had needed to seek help which contributed to feelings of being unprepared. Participants described previously delaying help seeking or waiting for someone to approach them directly which meant mental health problems could deteriorate.

Concerns about burdening others through disclosures relating to mental illness were apparent in both CYP and parental accounts. This combined with the aforementioned attitudes towards self-reliance and concerns about negative perceptions negatively impacted on help-seeking. These attitudes, however, could be challenged by positive experiences of help-seeking.

## Discussion

We undertook a qualitative study to compare the MHL of young people with anxiety and depression and their parents and to explore the impact of socio-cultural context on the experience of MHL. The study demonstrated limitations across all of the MHL components amongst both CYP and parents with CYP participants demonstrating limited awareness of their own conditions.

Cultural values were highly salient in participants' accounts relating to MHL and were useful in contextualising the themes that arose during data analysis. There were a range of views on what constitutes mental illness, professional support and self-management with identifiable and nuanced differences identified between the perceptions of CYP and their parents. This is likely to be important for those developing MHL interventions in terms of guiding the pitch, scope and context of future MHL programmes, policy and practices.

Culturally appropriate interventions have been defined as those that are built on the cultural values inherent to implementation contexts and include design and content features that adequately incorporate dominant cultural values and reflect the preferences and expectations of intended users (Marin, 1993). Future MHL interventions should, therefore, draw on recent evidence-based frameworks for cultural adaptation (Heim and Kohrt, 2019) and include all relevant stakeholders including young people in co-production (The Point of Care Foundation, 2013) to ensure maximal relevance, uptake and outcome.

The centrality of religiosity and spirituality within participant accounts reflected their dominance in wider belief systems and socio-cultural contexts inherent to Indonesia. As a consequence of this, religion was widely used for understanding the aetiology of and treatment for mental health problems and may represent a potential barrier in terms of accessing timely professional input (Muluk *et al*., 2018). One interpretation of the dominance of such beliefs along with the focus on personal responsibility within participants’ accounts is that they may underpin feelings of blame for the development of mental illness and contribute to self-stigma. This was due to such experiences often being considered a punishment from God or as a result of insufficient religious piety as described in both CYP and parental accounts. Such beliefs were more salient amongst parents compared to CYP which may reflect CYPs more limited exposure and earlier development stage. This represents an important target for future research and intervention in order to improve access to timely and effective treatments. In particular, understanding the de-implementation of existing practices in addition to the implementation of new evidence-based interventions should feature centrally in future research on the use of MHL interventions (Prasad and Ioannidis, 2014).

Notably, religious/spiritual healers and other lay network members were considered to be important sources of support and also an effective gateway to professional services. There appeared to be implicit trust in religious/spiritual healers whereas formal health services needed to demonstrate utility in order to increase confidence in efficacy. This highlights the potential for the development of interventions underpinned by holistic care approaches that optimally involve community members in enhancing access and delivery support, particularly given their unique capacity to negotiate the socio-cultural milieu in which mental health is experienced (Chowdhary *et al*., 2016). More widely, evidence suggests that communities in the WHO Southeast Asian region may more readily accept the preventive and curative services offered by lay workers (Dewi, 2011) and research indicates such approaches would also be acceptable to mental health professionals in Indonesia (Liem, 2020). Key questions regarding support for implementation, workforce training, incentivisation, regulation and governance require further exploration.

Global growth in mental health promotion and illness prevention may usefully benefit from targeted, population specific MHL initiatives embedded within broader programmes of stigma dilution. Policy and practice development in this field must remain cognisant of differences between individual and collective health cultures, and their potential requirements for different approaches. Young people's MHL in particular, is likely to be influenced by a myriad of social influences including to a greater or lesser degree, parental health beliefs and behaviours.

Our findings demonstrate that an individual's personal experience may be an important factor impacting MHL, such that exposure to positive treatment experiences may promote further help-seeking and reduce stigmatising attitudes across generations. Robust community engagement and involvement in intervention design, delivery and evaluation has the potential to raise awareness of the negative impacts of common mental health disorders, reduce stigma and increase demand for effective and accessible treatments (Fuhr *et al*., 2014; Crocker *et al*., 2018; Brooks *et al*., 2019b).

Participants described high levels of stigma towards those with mental illness within local communities that appeared to reflect wider societal views in Indonesia (Irmansyah *et al*., 2009; Susanti *et al*., 2020). Parents appeared to manage this stigma and potential threat to self by differentiating their children from those with more serious mental health problems. The latter were ‘othered’ in narratives and described in negative and stigmatising ways which appeared to be due to low levels of awareness of mental illness and mirrored the aforementioned socio-cultural context. Such conceptualisations are in stark contrast to the five key principles, or the ‘*Butir-butir*’ *Pancasila*, adhered to by the Republic of Indonesia since it gained independent status in 1945 namely the second principle of justice and civilised humanity (Rugebregt, 2019). Future intervention activities should attempt to harness synergies with these collectivist/communalistic values in order to maximise intervention reach and impact.

Initiating intersectoral support, for both community engagement and treatment delivery, offers one potential mechanism for optimising health returns. Both CYP and parents in our study acknowledged that teachers are likely to play an important role in the provision or reinforcement of MHL interventions. Peer support programmes facilitated in educational settings may also benefit young people, providing that both parties are adequately trained to reduce the risks of transmitting sub-optimal information exchange and/or perpetuating existing levels of bullying and stigma (Walker and Bryant, 2013).

Our study identified misconceptions and incorrect assumptions amongst parents and CYP, and towards each other. Despite CYP expressing a preference for familial support, they often reported not wanting to burden parents or not being listened to previously, which limited their confidence in this avenue of help seeking for the future. Parents often felt that their children would not wish to speak to them about their mental health and would prefer to talk to friends or teachers. These findings are in part likely to reflect the manifestation of respectful shame and reluctance to burden others (‘*isin*’ and ‘*sungkan*’) which is dominant in the Javanese culture (Geertz, 1959; Brooks *et al*., 2022). The contradiction in views also highlights considerable potential for sub-optimal communication within families, a finding further supported by parents not appearing to draw on children's lived experience to improve their own MHL. Family and community level interventions which adequately incorporate cultural issues to support children with mental health problems are likely to be important components in a portfolio of practices intended to promote effective help-seeking and self-management (Pedersen *et al*., 2019).

### Strengths and limitations

This study gains its strength from its use of qualitative methods underpinned by the Mental Health Literacy Framework (Jorm *et al*., 1997). This framework has since been expanded to include positive mental health (including resilience and salutogenesis) and stigma as well as self-management self-efficacy (Kutcher *et al*., 2015). This conceptual evolvement more fully reflects the burgeoning evidence base in relation to MHL and the growing recognition of social context in which mental health is experienced and help-seeking enacted (Kutcher *et al*., 2016)

The original framework was used for the purposes of this study but we remained cognisant of contemporary modifications as our study progressed. We included conceptualisations of positive mental health and stigma into our analyses to align with the conceptual evolution of MHL, providing an early lens through which to view, identify and target potential opportunities for action (Jorm, 2020).

The comparative approach allowed for the identification of similarities and differences in the accounts of young people and their parents which contributed to an in-depth understanding of MHL amongst these groups and the impact of cultural concepts, values and beliefs. The study further benefited from patient and public involvement including the contributions of co-lead author BP and the study advisory panels which ensured interpretations remained grounded in the experience of mental illness in Indonesia.

Data were not collected from other members of communities implicated by participants in the current study (professionals, spiritual/religious leaders and teachers) and future research would benefit from exploring the views of these stakeholders. Participants were self-selecting and were limited to those who expressed an interest in taking part and responded to direct invitation from health services, therefore, are likely to have better MHL than those who have not had contact with formal health services and more positive views about mental health services generally (Brooks *et al*., 2022). While participants were purposively sampled to ensure diversity in terms of gender, age and geographical location, future recruitment activities should endeavour to include those with experience of anxiety and depression who are not in contact with formal health services and those from other geographical areas to ensure their perspectives are represented.

## Conclusion

Robust efforts are needed to improve MHL in LMICs drawing on culturally appropriate approaches to reduce stigma and optimise timely and effective help-seeking.

## Footnotes

1 Culturally appropriate interventions are deemed to meet the following criteria: ‘(a) The intervention is based on the cultural values of the group, (b) the strategies that make up the intervention reflect the subjective culture (attitudes, expectancies, norms) of the group, and (c) the components that make up the strategies reflect the behavioral preferences and expectations of the group's members’ (Marin, 1993).

## Appendix 1: Interview schedules

### Parent interviews

Recognition

- What do you think the signs of mental health problems are? If unsure, prompt: do you think they affect how a person feels, such as how happy or sad they are? Aspects of their life affected such as school, work, family life or friendships.
- When did you first notice that your son/daughter might be experiencing a mental health problem? How did you know something was wrong? What did you do?
- What do you know about different types of mental health problem?
- What do the words ‘depression’ and ‘anxiety’ mean to you?

Risk factors/causes

- Why do you think some people have mental health problems? How do they come about? If unsure – suggest a few causes, e.g. stressful event, passed on through the family, supernatural causes.
- What do you think caused your child's depression and/or anxiety symptoms to start? When did it happen? Do you think there might be any other possible explanations? Is there anything that makes it better or worse?

Knowledge/beliefs about self-help interventions

- Is there anything that young people can do for themselves to look after their mental health? If unsure, prompt – what about talking to family, friends, taking part in sport?
- Does your child do anything to improve their mental health? How useful do you think that is? Does it work? Why?
- Can you help them? What do you do to help? How useful do you think that is? Does it work? Why?

Knowledge and beliefs about professional help

- Do you think professionals (e.g. doctors, psychologists, psychiatrists) can help people with mental health problems to get better?
- Do you think professionals have helped or could help your son/daughter? If so, how? If not, why not? Are professionals the best source of help? What would be the best source of help?
- What sort of help could/would help the most? If unsure –prompt: medicines, talking therapies, traditional healers/medicine, sports/social media, etc. Why?

Attitudes towards recognition/help-seeking

- If you were having problems with your mental health (such as feeling low or worried/anxious), would you try to get help? Would this be easy or hard, and why? Would anything get in the way of you trying to get help? What would help you to get help?
- What do the people around you (e.g. other members of your family, community/society) think about mental health problems?
- What was your reaction to realising your son/daughter might be having problems with their mental health? What did you do? Did you think it was important to seek help? Did anything get in the way of encouraging your son/daughter to seek help? What made it easier?

Knowledge of how to seek information

- If you wanted to know more about your child's mental health problem or about mental health in general, where would you find more information? Who would you ask/where would you look?

### CYP interviews

Recognition

- What do you think the signs of mental health problems are? If unsure, prompt: do you think they affect how a person feels, such as how happy or sad they are? Aspects of their life affected such as school or friendships.
- Do you think you would know if you, or someone close to you, were experiencing mental health problems? How would you tell?
- What do you know about different types of mental health problem?
- What do the words ‘depression’ and ‘anxiety’ mean to you? If not already covered – how would you recognise these problems?
- When did you first notice that you might have a mental health problem? How did you know something was wrong? Did you think your problems might be part of depression/anxiety?

Risk factors/causes

- Why do you think some people have mental health problems? How do they come about? *Prompt – ask about depression and anxiety specifically.*
- What do you think caused your mental health problems to start? When did it happen? Do you think there might be any other possible explanations for why your problems started? Is there anything that makes it better or worse?

Knowledge/beliefs about self-help interventions

- Is there anything that young people can do for themselves to look after their mental health? If unsure, prompt – what about talking to family, friends, taking part in sport?
- Do you do anything to improve your mental health? How useful do you think that is? Does it work? Why?
- How could you help other people who are experiencing mental health problems? Do you think you could help? How?

Knowledge and beliefs about professional help

- If you were experiencing a mental health problem, would you want to seek help? Why? Would you know how to get help? What would you do? Could anyone help you get support? E.g. accompanied by friend/family member
- Do you think professionals (e.g. doctors, psychologists, psychiatrists) can help people with mental health problems to get better? What about depression and anxiety in particular? If so, how? If not, why not? Are professionals the best source of help? What would be the best source of help?
- What sort of help would help the most? If unsure –prompt: medicines, talking therapies, traditional healers/medicine, sports/social media, etc.
- What sort of help has been most useful to you, if any?

Attitudes towards recognition/help-seeking

- If you were having problems with your mental health (such as feeling low or worried/anxious), would you try to get help? Would this be easy or hard, and why? Would anything get in the way of you trying to get help? What would help you to get help?
- What do the people around you (family, school friends, teachers, community) think about mental health problems?
- How easy or hard was it for you to tell someone that you were having problems? What/who helped you tell others? Did anything/anyone make it more difficult?

Knowledge of how to seek information

- How would you find out more about mental health if you had a question? Who would you ask/where would you look?
- If you wanted to know more about your depression or anxiety, where would you find more information?

## Appendix 2: Exemplary quotes to illustrate both convergent and divergent views between CYP and parents for each MHL component

**Table:**

Mental Health literacy framework component	Convergent views	Divergent views
Recognition	**Limited awareness of formal diagnostic categories:** *Q: What do you think mental health problem means?* *R: Errr…I don't know.* *Q: How about mental illness?* *R: I don't know…Maybe crazy people.* **A212, Jakarta CYP** **Description of anxiety and depression:** *People with anxiety always think or get worried about everything. Like when I was worried about how I was going to be placed, that was always on my mind, I had a headache and my heart was beating very fast, like when I was in a debate in class.* **A210, Jakarta CYP** **Differentiation between common mental health problems and more severe mental illnesses.** *Do you think there's any difference between someone with a mental health problem and someone with mental illness?* *R: They're different, in my opinion.* *Q: How are they different?* *R: Like my child, she just has a mental health problem, not like some crazy person with a psychological problem. Her behavior is also different from people with severe psychological problems.* **C320, Magelang, parent.** **Mental illnesses as a form of abnormality:** *The crazy ones are the ones who are already severely mentally ill, the ones who yell at nothing and stuff. But I think, you can see someone's mental health by looking at how they can't control their emotions, and maybe you can see it from their behavior, like doing something illogical.* **A316, Jakarta, Parent.** *Q: How about poor mental health?* *R: Err, different from the others, not normal.* **A212, Jakarta, CYP**	**Recognition of CYP mental illness:** *R: I don't remember…I don't know. I know it's been a while. I've had it since at least two years ago.* *Q: How did you know that something was wrong?* *R: Because someone told me.* *Q: Oh, someone told you…Who told you?* *R: Mom. She said that I was sick.* **B209, Bogor, CYP** *What do you think triggered your child's mental health problem?* *R: Well, just from her history.* *Q: When did that happen?* *R: I think since elementary school…It started when she started elementary school, but it was very apparent in second grade.* **C317, Magelang, parent.** **Indicators of mental illness:** Q: How did you know that something was wrong with yourself? *R: Because I didn't know that I had mental health* [*sic*], *I didn't know. After listening to this now I know about mental health problems.* *Q: What's the mental health problem that you have?* *R: Well, I get angry a lot.* *Q: Errr, so that's a mental health problem?* *R: Errr, yeeees.* **B210, Bogor CYP.** *Q: How did you know there was something wrong with them?* *R: Like I mentioned, they stopped taking care of themself. They rarely showered. Their daily schedule started to become all over the place, and they had no fear nor respect toward us as their parents. They started to avoid their friends, like they were the only person in the world. They'd lock themself in their room. As for their relationship with their teachers…They didn't want to go to school anymore. They'd ignore the rules.* **C322, Magelang, parent.**
Causes	**Individual failure:** *Q: What other reason can cause it, other than their surrounding?* *R: Their family, like me now, I always get too sensitive and think negatively.* **B213, Bogor, CYP** *I: So it started in third grade?* *R: That was when they started to get lazy, they didn't want to go to school…* **C322, Magelang, parent** **Biological and developmental causes:** *I: How could that happen? For example, can it be hereditary?* *R: Yes, from Dad…Dad also has mental health problems*. **A213, Jakarta, CYP** *R: Our heads felt heavy, we had headaches, our bodies were hurting…When your mental state isn't healthy, your body wouldn't be healthy either. We went to do a general check-up, and it turned out that there was nothing wrong with us* [*physically*]. *It affected our school life because we would rather be alone, we weren't confident in ourselves, and when we did go to school, we couldn't focus on the lessons. We wanted to run away from reality because we felt stressed.* **B315, Bogor, parent** **Trauma:** *I: Was there anything else that caused your child's mental health problem? If yes, elaborate.* *R: There was physical violence from my husband, and a lot of throwing things into the floor.* **C317, Magelang, parent**	**Parents more likely to identify poor parenting and family relationships as causes:** *I: What do you think triggered your child's mental health problem?* *R: Maybe because we didn't understand how to raise a child well.* **C317, Magelang, parent** **CYP more likely to identify bullying and punishment from teachers as a cause:** *I: How often did they do that?* *R: Almost every month, every week. They didn't understand my condition that made me unable to hang out too often. I stayed home more often, but they'd always urge me to come and hang out. They'd physically bully me by pushing me*. **B210, Bogor, CYP**
	**School/relationships with peers:** *R: They had a problem with a teacher as well. They felt that the teacher judged them like they were, well, a bad kid who wouldn't listen and would often cause problems. They're holding a grudge because of that.* **C322, Magelang, parent** **Environmental causes** **Religious or supernatural causes:** *I: Trial? What kind of trial?* *R: There's a lot of them, there's always a trial from God for us, it can come from ourselves, from our neighbors, family, surrounding, or our school friends.* **B211, Bogor, CYP** *I: In your experience, why do you think someone is depressed or anxious?* *R: (stays quiet, reluctant to answer)* *I: Was it because a bad experience? Did you have a spiritual experience, like a djinn bothering you?* *R: A djinn was bothering me. I was chased by a ghost once and I was scared.* **B212, Bogor, CYP**	
Help-seeking knowledge	**Individual responsibility for self-management** *But we, as parents, also need to give out advice as well. Doctors, therapists can help you, but the rest is you having to want to get yourself better. That's why we as parents need to control our kids. We need to keep an eye on them, even if they say that they don't want us to do that.* **B315, Bogor, Parent.** *Q: What kind of help is the most helpful?* *R: Reminding me to pray more, you need to be…* *Q: You need to be what?* *R: You need to be in control of yourself.* **B209, Bogor, CYP.** **Valued activities:** *I try to busy myself by socializing, studying, surfing websites. These activities give me a purpose for the future and make me want to get out of the black hole* **A210, Jakarta, CYP.** *She likes to sew, and also feed stray cats. That's something I encourage her do, so that she knows how it's like to care about something. It helps a lot. By doing things she likes, it decreases the time she used to use to think and remember about the things that traumatized her. It's something I encourage her to do so that she doesn't keep remembering her past, and so far it's worked.* **B315, Bogor, parent.** **Value of lay networks** *Q: What kinds of things do you think can help young people maintain their mental health?* *R: Bettering themselves and asking questions.* *Q: Asking questions to whom?* *R: Parents, teachers, because they can help us. They've experienced it before.* **C214, Magelang, CYP** **Value of professional support related to pas experience** *I: Do you think professionals can help your child? If yes, how? If not, why?* *R: They can give us, the parents, some advice because as people who are not an expert, we don't really understand, and we'd just judge our kids without knowing what caused it. The professionals help us to open our eyes, that there are things inside your child that makes them like this. They didn't become a bad kid out of nowhere, but there's something inside. The professionals make us better at handling our child*. **C322, Magelang, parent** *R: Well, I can't really say it's the best because I haven't seen any results of it yet. When it's shown results, only then can I say if it's the best or not. It hasn't shown any results yet, so I don't know if it's the best or not.* **B316, Bogor, parent** **Value of trusted technology based information:** *For more comprehensive information, I would look on Google.* **B321, Bogor, parent**	**Parents more likely to foreground religious and spiritual activities for self-help** *I support her by telling her that, well, if she feels* [*the anxiety coming*], *then she should say astagfirullahaladzim, Allahu akbar (T/N: I seek forgiveness to Allah and repent towards Him, Allah is great). We need to remember that when we need help, the first one we should ask is Allah. We need to chant, la hawla wala quwwata illa billahil adzim (T/N: There is no power and no strength except with Allah). That's what she usually does at home. She would read the Quran. It's better to read the Quran and do the salat prayers rather than doing nothing or just daydreaming.* **B316, Bogor, parent.** *Q: What kinds of things do you think can help young people maintain their mental health?* *R: Like I said earlier, going to the school in the morning, going to the mosque in the afternoon. That's what I want them to do, doing a group Quran reading, but they don't want to.* **C318, Bogor, parent.** **Parents more likely to feel that CYP would be reluctant to speak to parents about mental health difficulties:** *R: I usually would ask someone else's help. We don't understand. Sometimes people – with their own parents, they…How do I say it…they're more closed off. I respect her when she chooses to talk to someone else, whether it's a guy or a girlfriend, rather than with her own parent.* **C320, Parent, Magelang** **Whilst rare, CYP were more likely to highlight negative aspects or help-seeking from lay networks:** *I like to put down my feelings there. I like talking about my feelings to someone else like my mom, but my mom doesn't want to* [*listen to it*]. *Whenever I talk about my feelings, I get scolded.* **C209, Magelang, CYP.** *When I'm with friends I get more tired, I feel more intimidated.* **B210, Bogor, CYP** **Parents viewed of professional support extended to spiritual healers in which there was implicit trust:** *R: By giving them some medicine…Especially when we can't handle them…Going to the doctor is my last alternative…I also have a spiritual teacher, and I consult them a lot…**A314, Jakarta, parent*** **CYP more heavily reliant on family and technology whereas parents have a broader repertoire of support. Parents foregrounded religious support:** *I'd go to this hospital and ask a doctor directly. I can't use the internet like that. I only use my phone for calls*. **C319, Magelang, Parent** *Q: How do you look for more information if you want to know more about mental health?* *R: Religion.* *Q: Who will you ask/where will you try to get information from?* *R: I'd ask for help from the ustadz (T/N: Islamic teacher).*
		*Q: What does the ustadz usually tell you?* *R: A religious speech.* **C214, Magelang, CYP** *From my own experience, and I'd watch TV shows about mental health, and attend religious events led by the Islamic teacher talking about mental health.* **C324, Magelang, Parent** *Q: How do you look for more information if you want to know more about your child's mental health?* *R: Reading books and watching movies.* **A316, Jakarta, parent**
Beliefs about facilitators and barriers.	**Anticipation of negative attitudes and stigma:** *It's hard because I'm afraid that they'll mock me. Sometimes I get embarrassed to talk to someone else.* **A210, Jakarta, CYP** *The doctor who treated him did say that…Ma'am…Your kid, he would either die or become an idiot…If he doesn't become an idiot…When he grows up he would have problems…***B317, Bogor, parent** **Value of lay networks:** *Alhamdulillah, thank God the almighty, my surrounding helped me a lot, and my friends gave me advice as well, like try to take your child to this place or that place.* **C319, Magelang, Parent** **Concerns about burdening others:** *Q: If you yourself feel worried, anxious, or down, will you seek help? Like A did. Or would you consult a doctor here, or would you do something else?* *R: More often I'd keep it to myself.* *Q: Have you ever gone here and consult someone about your problem, not your child's?* *R: No. I'd just cry to myself.* *Q: So you talk more often to your own mother?* *R: I used to. But now my mother is very old, so I don't want to burden her anymore.* **C320, Magelang, parent** *It's hard, Mom is busy.* **C213, Magelang, CYP** **Value of positive experiences of help-seeking:** *Q: Does it feel very bad now?* *R: If I talk about it or share it with someone, I would feel lighter.* *Q: How does it feel like right now? Like carrying some stones? Then just put the stones away, hehehe.* *R: The stones are important, so I can't put them away. So I need to talk about them or share them so I'd feel lighter.* **B213, Bogor, CYP** *Q: If you have a mental health problem, will you seek help? Why?* *R: Yes, so that it won't happen again.* **C216, CYP, Magelang.**	**CYP described lack of confidence in expressing emotions to facilitate help-seeking:** *Q: If you have a mental health problem, will you seek professional help?* *R: Errr…No.* *Q: Why?* *R: Well, I'll wait until someone comes to help me.* **A212, Jakarta, CYP** *I think it was because they felt reluctant to talk about it, and when they were feeling very stressed they wouldn't talk to anyone that they were feeling that way. But if they had said that they weren't feeling okay, maybe others might've been able to help them, and their condition wouldn't have been worse* **A315, Jakarta, parent**
